# Significance of debriefing methods in simulation-based sedation training courses for medical safety improvement in Japan

**DOI:** 10.1186/2193-1801-3-637

**Published:** 2014-10-28

**Authors:** Nobuyasu Komasawa, Takuro Sanuki, Shunsuke Fujiwara, Masanori Haba, Ryusuke Ueki, Yoshiroh Kaminoh, Toshiaki Minami

**Affiliations:** Department of Anesthesiology, Osaka Medical College, Daigaku-machi 2-7, Takatsukishi City, Osaka, 569-8686 Japan; Department of Dental Anesthesiology, Nagasaki University, Sakamoto 1-7-1, Nagasaki City, Nagasaki, 852-8588 Japan; Department of Anesthesiology, Wakayama Red Cross Hospital, Komatsubara 4-20, Wakayama City, Wakayama, 640-8558 Japan; Department of Anesthesiology, Hyogo College of Medicine, Mukogawa-cho, Nisinomiya City, Hyogo, 663-8501 Japan; Division of Anesthesiology, Social Insurance Kinan Hospital, Shinjo-cho, Tanabe City, Wakayama, Japan

**Keywords:** Sedation and analgesia, Simulation training, Debriefing, Medical safety

## Abstract

Based on the American Society of Anesthesiologists’ Practice Guidelines for Sedation and Analgesia by Non-Anesthesiologists (ASA-SED), a sedation training course aimed at improving medical safety was developed by the Japanese Association for Medical Simulation in 2011. This study evaluated the effect of debriefing on participants’ perceptions of the essential points of the ASA-SED.

A total of 38 novice doctors participated in the sedation training course during the research period. Of these doctors, 18 participated in the debriefing group, and 20 participated in non-debriefing group. Scoring of participants’ guideline perceptions was conducted using an evaluation sheet (nine items, 16 points) created based on the ASA-SED.

The debriefing group showed a greater perception of the ASA-SED, as reflected in the significantly higher scores on the evaluation sheet (median, 16 points) than the control group (median, 13 points; p < 0.05). No significant differences were identified before or during sedation, but the difference after sedation was significant (p < 0.05).

Debriefing after sedation training courses may contribute to better perception of the ASA-SED, and may lead to enhanced attitudes toward medical safety during sedation and analgesia.

## Background

Sedation is performed in various medical procedures and examinations. Because most of these procedures are performed in environments which lack monitoring and emergency medication, inappropriate sedation causes various challenges about medical safety (Desai 2008).

In 1993, the American Society of Anesthesiologists published the Practice Guidelines for Sedation and Analgesia by Non-Anesthesiologists (ASA-SED), which was updated in 2002. These guidelines set forth recommendations and cautions for non-anesthesiologists to ensure safe and effective sedation and analgesia. High quality, safe sedation requires preoperative patient examination, confirmation of fasting time, appropriate monitoring, adequate emergency equipment, compliance with drug administration principles, and validation of discharge criteria. Practitioners are also cautioned to be aware that the level of sedation changes under different circumstances (Practice Guidelines for Sedation and Analgesia by Non-Anesthesiologists 96).

In 2011, the Japanese Association for Medical Simulation developed a simulation-based sedation training course (SEDTC), with the goal of improving the safety of sedation and analgesia performed by non-anesthesiologists. Participants in the course are expected to demonstrate (1) appropriate preparation for sedation, (2) appropriate management of drug-induced hypoxia and/or shock, and (3) effective communication skills (Komasawa et al.).

Debriefing and reflection is a vital component of simulation training. Feedback associated with debriefing gives learners the opportunity to think critically about their performance. Learners can deconstruct events and errors that unfold during the scenario, and acquire new information to improve subsequent practice by appropriate debriefing (Aronson 2011; Sawyer et al. 2013).

We hypothesized that debriefing would enhance the perceptions of essential points of the ASA-SED, as well as attitudes toward medical safety and sedation during simulation-based SEDTCs. To this end, we evaluated the effect of debriefing on participants’ perceptions of the essential points of the ASA-SED.

## Methods

This study was approved by the Institutional Review Board of Hyogo College of Medicine (No.1317), and participation in the course was considered as consent to participate in the study.

Contents of the course are summarized in Table 1. The SEDTC included the following: a lecture introducing the ASA-SED, a discussion and card simulation about several sedatives and analgesics, basic airway management training using a manikin, and scenario-based training using a simulator. A 60-minute lecture summarizing the guidelines was also conducted. After training, participants engaged in a card-based discussion on characteristics and titration methods for sedative and analgesic medications (45 minutes), hands-on emergency airway management practice (including insertion of supraglottic devices such as laryngeal and ventilation masks, 45 minutes), and simulated scenario training (60 minutes). Usually 10 participants per instructor participate in the SEDTC. In the first twelve courses, we did not apply debriefing and discussion and we evaluated the effect of debriefing from 13^th^ courses.

**Table 1:** **Content of simulation-based sedation training course**

	Content
Lecture	Introduction of the summary of guideline about sedation and analgesia for non-anesthesiologists by American Society of Anesthesiologists.
Discussion and training utilizing card	Group discussion and lecture about classification, mutual effect, reversal agent about several sedatives and analgesics utilizing card.
Hands on training for basic airway management utilizing manikin	Evaluation of respiratory status (respiratory pattern, rate, SpO_2_ monitoring etc), method for oxygen administration, basic airway management including bag-valve-mask and supraglottic devices utilizing manikin.
Scenario-based training utilizing simulator	Simulation based training about sedation including preoperative evaluation, monitoring, emergency response, and criteria of discharge utilizing simulator

SEDTCs were conducted with or without debriefing in the 13^th^, 16-19^th^, and 24^th^ SEDTCs. Debriefing was performed or not in the last scenario-based training utilizing simulator. Conduct of debriefing was decided according to the envelope method. Instruction with debriefing was included in the 13^th^, 17^th^, and 19^th^ courses, but not in the 16^th^, 18^th^, and 24^th^ courses. The level of perceptions was evaluated for novice doctors (initial trainee doctors) who had less than two years of clinical experience in the Japanese medical system. Their clinical experience is 38 initial trainee doctors (22 males and 16 females, 25.3 ± 2.4 years old) is 0.9 ± 0.4 years. They attended SEDTC for the learning of principles/basics of effective and safe anesthesia.

Debriefing was provided in an ask-tell-ask approach, as follows: 1) students were asked to provide self-reflections about perceived strengths and areas for improvement; 2) faculty provided tailored responses that included specific feedback points based on observations; and 3) faculty asked students about the major take-home points from the debriefing and addressed any additional questions. In the non-debriefing group, participants were given feedback about appropriate action from the instructor.

An evaluation scenario was conducted immediately after the course. Scoring of participants’ guideline perceptions was conducted using an evaluation sheet (nine items, 16 points) that was created based on the ASA-SED (Table 2). We developed this checklist to reflect the each important points of ASA-SED. To avoid bias, the same instructor evaluated the score. The instructor passed the paper to participants which following scenario is written. We did not use this checklist during scenario training during SEDTC.

**Table 2:** **Evaluation sheet based on ASA-SED applied in this study**

Evaluation item		Points
**Before sedation**		
**1**	Confirmation of food and drink intake	1
**2**	Mounting the monitor (oxygen saturation meter is required)	1
**During sedation**		
**3**	Appropriate sedative drug administration	1
**4**	Evaluation of sedation level	1
**5**	Proper airway management (points added according to the number of items completed)	1–3
	・oxygen administration ・mandibular elevation ・insertion of air way tool	
**6**	Appropriate drug (administration of analgesics for pain)	1
**After sedation**		
**7**	Consideration for re-sedation (2 h of observation after administration of antagonist drugs)	1
**8**	Evaluation in recovery room (points added according to the number of items completed)	1–3
	・record consciousness level at regular intervals・record vital signs at regular intervals・exclusive work in the recovery room	
**9**	Evaluation during hospital discharge (points added according to the number of items completed)	1–4
	・confirmation of consciousness level・confirmation of vital signs・confirmation of patient’s assistant (or attendant)・confirmation of methods for contact	

The evaluation scenario was as follows:

“A 50-year-old woman with body height of 150 cm and body weight of 40 kg underwent removal of multiple colon polyps via lower gastrointestinal endoscopy under sedation. The surgery ended 30 minutes earlier than originally planned. The patient was allergic to both eggs and soybeans. No specific problems were detected during preoperative examination. The patient was instructed to fast, without eating for six hours or drinking for two hours prior to surgery. All points to be confirmed and procedures to be conducted from the time of the patient’s admission until her discharge were spoken aloud. Please go through treatment under sedation. Please ask the instructor if you have any questions regarding the patient’s condition.”

In addition to evaluating participant perceptions of the guidelines, the satisfaction rate of individuals who participated in the course was evaluated using a 100 mm visual analog scale (VAS).

The Mann-Whitney U test was used to compare evaluation scores and the unpaired t-test was used to evaluate VAS scores for course satisfaction. P < 0.05 was considered statistically significant.

## Results

A total of 38 novice doctors participated in the SEDTC during the research period, with 18 trainees in the debriefing (D) group, and 20 in the non-debriefing (N) control group. The D group showed significantly higher evaluation scores than the N group, with median scores of 16 points and 13 points, respectively (p < 0.05). No significant differences were identified before or during sedation, but the difference after sedation was significant (p < 0.05). VAS scores for course satisfaction were significantly higher in the D group than in the N group (D group, 90.2 ± 7.0 mm; N group, 85.0 ± 7.9 mm; p < 0.05) (Table 3).

**Table 3:** **Comparison of evaluation score between Debriefing and Non-debriefing group**

	Debriefing group (n = 18)	Non-debriefing group (n = 20)	P value
Before Sedation	2 [1,2]	2 [1-2]	0.35
During Sedation	6 [4-6]	5 [4-6]	0.18
After Sedation	8 [5-8]	6 [4-8]	0.003
Total score	16 [13-16]	13 [10-15]	0.003

Data are presented with median [range], Total score is sum of three phases.

## Discussion

Sedation for procedure is administered to patients from various clinical situations by diverse practitioners (Lichtenstein et al. 2008; Daniels et al. 2013). Sedation includes a continuum of consciousness, progressing through mild, moderate, deep sedation, to general anesthesia. We could not always predict how an individual patient respond to the sedation performed. Though target levels of sedation have been defined, actual depth levels can easily fluctuate. Thus, the ASA-SED recommends that patients be continuously monitored by medical staff who does not directly involve in the procedure. The guideline also recommend that the sedation provider be qualified to rescue patients whose levels of sedation become deeper than initially intended (Practice Guidelines for Sedation and Analgesia by Non-Anesthesiologists 96). One closed claims analysis showed that the most common complication during procedural sedation is respiratory depression due to over-sedation (Desai 2008).

The purpose of the ASA-SED is to allow non-anesthesia doctors to provide their patients with the benefits of sedation while minimizing associated risks. Sedation and analgesia provide two general benefits. First, appropriate sedation allows patients to tolerate unpleasant procedures by relieving discomfort, anxiety, or pain. Second, sedation may expedite procedures that are not particularly uncomfortable, but that require patients to remain still in children and uncooperative adults. However, sedation sometimes result in cardiac or respiratory depression, which should be rapidly detected and appropriately managed in order to minimize the risks of adverse events (Moran et al. 2013). Furthermore, inadequate sedation may induce unnecessary patient discomfort or injury, if associated with a lack of cooperation or adverse physiologic and psychological responses to stress. Providing an appropriate level of sedation is sometimes difficult for non-anesthesiologists, and educational techniques such as simulation-based SEDTCs are important for improved safety management (Practice Guidelines for Sedation and Analgesia by Non-Anesthesiologists 96).

The positive effect simulation training can be maximized when it is closely aligned with the clinical situation or environment (Lorello et al. 2014). For this purpose, simulators have evolved from classic models to simulated virtual worlds (Abdelshehid et al. 2013). Effective debriefing and feedback have been identified as essential components of simulation-based training (Sawyer et al. 2012; Issenberg et al. 2005; McGaghie et al. 2010). The facilitation of simulation debriefings can provide novice doctors opportunity to learn various points by identifying and reflecting on the errors and findings (Naik et al. 2013).

Without debriefing, the level of post-care perceptions was found to be lower, particularly the level of perceptions following sedation. This simulation course provided an influential educational effect via debriefing, and resulted in a high rate of satisfaction. Especially, the consideration toward after sedation showed a marked improvement. Furthermore, debriefing provided not only an influential educational effect, but also a high rate of satisfaction.

Among the sedation phase, debriefing did not significantly improve the evaluation score on ‘before sedation’ and ‘both sedation’. In contrast, debriefing significantly improved evaluation score on ‘after sedation’. This result suggests that initial trainee doctors are difficult to imagine and cultivate their attitude about crisis after sedation. As postoperative complication is various, it may be difficult for novice doctors with small clinical experience about the safety management after sedation. Debriefing may debriefing may help initial trainee doctors for imagine and develop their idea toward post procedure sedation safety (Chung et al. 2013; Schmidt et al. 2013; Allen 2013).

In the present study, we did not evaluate their perception level before SEDTC. In their next study, we would like to consider including a 0-level questionnaire before the SEDTC in order to find out the level of knowledge prior to the study and evaluate the education effect of the course.

Anesthesiologists, who generally have deep understanding of respiratory physiology, airway management, and sedation and analgesia administration, should lead efforts to improve hospital-wide safety management. To achieve this, in addition to educating medical staff about sedation techniques, the sedation system must be reconstructed. For example, those who develop safety systems should also be obliged to monitor and determine standards for discharge. A deep understanding of sedation principles and safety management is important, as is the development of a system for hospital-wide medical safety (Kiersma et al. 2011). We anticipate that SEDTCs will contribute to this goal through the training of non-anesthesiologists.

## Conclusions

Debriefing in SEDTCs may contribute to better perceptions of ASA-SED principles, and may lead to enhanced attitudes toward medical safety during sedation and analgesia.

## Abbreviations

ASA-SED: American Society of Anesthesiologists’ Practice Guidelines for Sedation and Analgesia by Non-Anesthesiologists
SEDTC: Sedation training course
VAS: Visual analog scale.
